# The emerging role of CaMKII in cancer

**DOI:** 10.18632/oncotarget.3955

**Published:** 2015-04-29

**Authors:** Yan-yang Wang, Ren Zhao, Hong Zhe

**Affiliations:** ^1^ Department of Radiation Oncology, General Hospital of Ningxia Medical University, Yinchuan, Ningxia, China; ^2^ Cancer Institute, Ningxia Medical University, Yinchuan, Ningxia, China

**Keywords:** Ca2+/calmodulin-dependent protein kinase II (CaMKII), cancer, cell cycle, therapeutic target, CaMKII inhibitor

## Abstract

Ca2+/calmodulin-dependent protein kinase II (CaMKII) is a multifunctional serine/threonine kinases best known for its critical role in learning and memory. Recent studies suggested that high levels of CaMKII also expressed in variety of malignant diseases. In this review, we focus on the structure and biology properties of CaMKII, including the role of CaMKII in the regulation of cancer progression and therapy response. We also describe the role of CaMKII in the diagnosis of different kinds of cancer and recent progress in the development of CaMKII inhibitors. These data establishes CaMKII as a novel target whose modulation presents new opportunities for cancer diagnosis and treatment.

## INTRODUCTION

Calcium ion (Ca2+) is a ubiquitous intracellular signal responsible for a broad range of cellular events, such as cell growth, cytoskeletal organization, regulation of synaptic transmission, and Ca2+ homeostasis [1-3]. The Ca2+/calmodulin (CaM)-dependent protein kinases (CaMKs) are multifunctional serine/threonine kinases whose activity are regulated through Ca2+ signaling [4]. Recent studies demonstrated that high levels of different isoform of CaMK, especially for CaMKII, expressed in several cancers such as lung [5], breast [6], prostate [7] and colon cancer [8]. CaMKII phosphorylates nearly 40 different proteins, including enzymes, ion channels, kinases, and transcription factors [9, 10] and plays a critical role in the regulation of proliferation, differentiation and survival of various cancer cells [5-8]. In this review, we will focus on the structure and biology properties of CaMKII, including the roles of CaMKII in the regulation of cancer proliferation and therapy response. The role of CaMKII as a biomarker in cancer diagnosis and the application of CaMKII inhibitors in cancer research will also be discussed in this review.

## STRUCTURE AND ACTIVATION OF CAMKII

**Figure 1:**
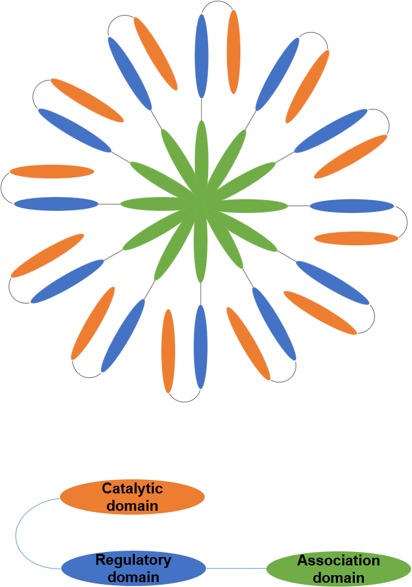
Schematic depiction of the Ca2+/calmodulin dependent protein kinase II (CaMKII) structure CaMKII holoenzyme is a dodecamer (top) and each monomer has the association domain, regulatory domain, and catalytic domain (bottom).

CaMKII is expressed as a multimeric protein, which typically comprised of 12 subunits in most commonly observed physiological conditions [11]. Each of these subunits has an N-terminus catalytic domain, followed by a regulatory domain, and a C-terminus association domain responsible for multimerization (Figure 1). Like other kinases, the catalytic domain of CaMKII has an ATP-binding pocket that creates a microenvironment to lower the energy required to hydrolyze ATP, enhancing the rate of transfer for the γ phosphate from ATP to a target S/T and ejecting ADP [12]. The regulatory domain of CaMKII has a C-terminus Ca2+/CaM binding region and an N-terminus autoinhibitory region [13]. The autoinhibitory region contains most of the elements that are critical for regulation of CaMKII activity, including the post translational modification (PTM) segment for phosphorylation, O-linked N-acetylglucosamine (O-GlcNAC) modification, and oxidation [9, 14].

There are four different CaMKII genes, and each gene encodes a distinct CaMKII isoform (β, γ, and δ). All CaMKII isoforms appear to share common regulatory mechanisms and protein targets but differ in tissue distribution [15]. Under resting conditions, the catalytic domain is constrained by the autoinhibitory sequences on the regulatory domain, thereby inhibiting the activity of the enzyme [12]. When intracellular Ca2+-levels periodically rise during the cellular Ca2+-transient, Ca2+ binds to CaM and activates CaMKII by binding to the regulatory domain. The activation leads to the phosphorylation of adjacent CaMKII subunits at Thr286 ( for the α isoform) or at Thr287 ( for the β, γ, and δ isoforms). The phosphorylation of Thr287 has at least two effects on CaMKII. The binding affinity of CaM for the CaMKII regulatory domain increases by more than 1000-fold. Additionally, the negatively charged phosphate group at the Thr287 site precludes reassociation of the catalytic and regulatory domains, preventing autoinhibion even if Ca2+ falls and CaM dissociates from CaMKII. The autonomous activation of CaMKII by Thr287 phosphorylation will persist until the phosphate group is removed by a protein phosphatase [10, 16, 17].

Elevated reactive oxygen species (ROS) level upregulates CaMKII through direct and indirect ways. The regulatory domain of CaMKII contains a pair of redox-sensitive amino acids (Cys280/Met281 in the α isoform, Met281/Met282 in the β, γ, and δ isoforms) that can be oxidized when exposed to elevated levels of oxidative stress. Like Thr287 autophosphorylation, Met281/Met282 oxidation prevents reassociation of the catalytic and regulatory domains even in the absence of Ca2+/CaM binding either [10, 18]. And also oxidation may increase the sensitivity of CaMKII to activation by Ca2+/CaM and the abundance of Thr287-autophosphorylated CaMKII by inactivating phosphatases [12].

**Figure 2:**
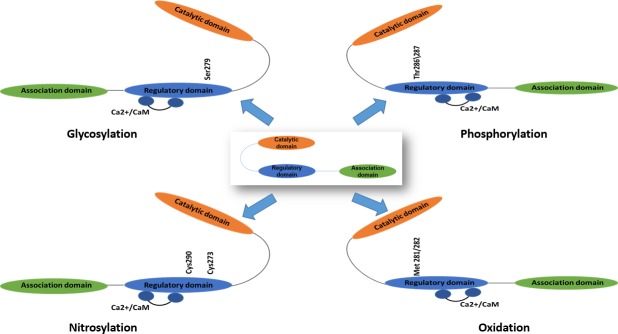
The activation mechanisms of Ca2+/calmodulin dependent protein kinase II (CaMKII) CaMKII could be activated by phosphorylation, oxidation, nitrosylation and glycosylation.

The underlying mechanisms for CaMKII activation during hyperglycemia and diabetes through the addition of an O-GlcNAC modification was found in recent study. O-GlcNAc modification of CaMKII at Ser279 activated CaMKII autonomously, creating molecular memory even after Ca2+ concentration declines [19]. In addition, CaMKII can be activated via nitric oxide (NO)-dependent nitrosylation of Cys273 or Cys290, the exact mechanism is still unknown at present [20] (Figure 2).

## THE ROLE OF CAMKII IN CANCER PROGRESSION

### Cell proliferation

Some studies have implicated CaMKII as an important player in cancer cell proliferation. The pharmacologic inhibition of CaMKII in MG-63 and 143B human osteosarcoma cells by KN-93, a chemical inhibitor of CaMKII, resulted in an 80 and 70% decrease in proliferation, respectively. The in vivo administration of KN-93 to mice xenografted with human osteosarcoma cells significantly decreased intratibial and subcutaneous tumor growth. And the inhibitory effect of CaMKII was associated with increased p21^CIP/KIP^ gene expression, protein levels, and decreased the phosphorylation of retinoblastoma (Rb) protein and E2F transactivation [21]. In normal and neoplastic B-lymphoid cells, suppression of CaMKII prevented the excessive B-cell activating factor (BAFF) -induced aggressive B-cell malignancies. It appeared that human soluble BAFF (hsBAFF)-mediated cell proliferation and survival was Ca2+-CaMKII-dependent [22]. In chronic myeloid leukemia (CML), cell proliferation specifically depends on activation of the CaMKIIγ isoforms. CML cells undergoing terminal differentiation or growth arrest display a marked reduction of CaMKIIγ autophosphorylation. Inhibition of CaMKIIγ resulted in the suppression of myeloid leukemia cell proliferation. The inhibitory effect of CaMKIIγ related to multiple signaling pathways, including mitogen-activated protein kinase (MAPK), signal transducer and activator of transcription 3(Stat3)/Stat5, and glycogen synthase kinase 3β (GSK3β)/β-catenin pathways [23]. Recently, Monaco et al. [24] reported that inhibition of CaMKII activity resulted in an upregulation of CaMKIV mRNA and protein in myeloid leukemia cell lines. Conversely, expression of CaMKIV inhibited autophosphorylation and activation of CaMKII, and elicited G_0_/G_1_ cell cycle arrest, impairing cell proliferation. These data reveal a novel cross-talk between CaMKII and CaMKIV and suggest that CaMKII suppresses the expression of CaMKIV to promote leukemia cell proliferation. CaMKIIγ promoted the cell proliferation via direct activation of nuclear factor kappa-light-chain-enhancer of activated B cells (NF-κB) and multiple oncogenic signaling pathways in non-small cell lung cancer (NSCLC) was found by Chai et al. CaMKIIγ could phosphorylate IκBα kinase β (IKKβ) at Ser177/181 and functioned as a mediator of IKKβ activation in NSCLC. In the meanwhile, CaMKIIγ could directly or indirectly upregulate multiple signaling pathways such as extracellular signal-regulated kinase 1/2 (Erk1/2), protein kinase B (Akt1), Stat3, and β-catenin and involve in regulating the survival and proliferation of NSCLC cells [5]. In papillary thyroid carcinoma (PTC), CaMKII is activated by BRaf^V600E^, Ras, and by RET/PTC. The activation of CaMKII subsequently leads to Erk activation and cell proliferation. Inhibition of CaMKII attenuates Erk activation and DNA synthesis in PTC cells [25]. Furthermore, the cell proliferation effects of CaMKII depend on the phosphorylated sites was found by Hoffman et al. [26]. They showed that the overexpression of a wild-type or Thr286 phosphomimic form of CaMKII increased proliferation rates of neuroblastoma and breast cancer cells, whereas overexpression of a Thr253 phosphomimic form significantly reduced proliferation rates in these cells.

### Cell cycle effects

**Figure 3:**
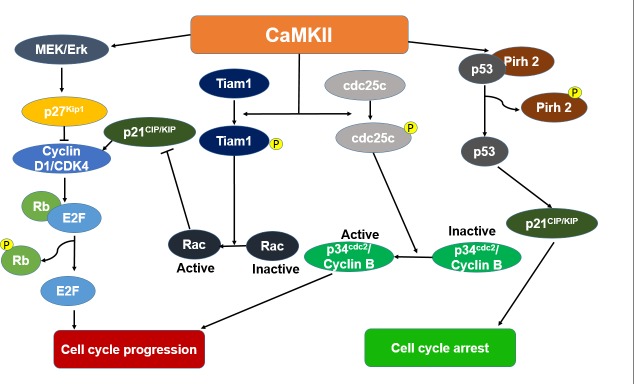
The proposed mechanisms depicting the cell cycle effect of Ca2+/calmodulin dependent protein kinase II (CaMKII) in cancer CaMKII activates cyclin-dependent kinase inhibitor 1B (p27^Kip1^) via the mitogen-activated/extracellular regulated kinase (MEK)/extracellular signal-regulated kinase (Erk) pathway. Activated p27^kip1^ will then inhibit cyclin-dependent kinase 4 (Cdk4)/CyclinD1 complexes, and therefore retinoblastoma (Rb) phosphorylation and E2F activation, induce cell cycle progression. CaMKII phosphorylates T-lymphoma and metastasis gene 1 (Tiam1) and then increases the activation of ras-related C3 botulinum toxin substrate 1(Rac1). The activation of Rac1 inhibits the expression of p21^CIP/KIP^. This leads to a cell cycle progression. CaMKII increases the phosphorylation of cell division cycle 25c (cdc25c) and actives p34^cdc2^/Cyclin B, causes the cell cycle progression. CaMKII suppresses cell cycle progression by stabilization of p53 via CaMKII-dependent phosphorylation of RING-H2 type E3 ligase (Pirh2).

CaMKII involves in the cell cycle control with a complex manner and associates with multiple cell signaling pathways (Figure 3). In colon adenocarcinoma cells, CaMKII activates mitogen-activated/extracellular regulated kinase (MEK)/Erk, which is responsible for the phosphorylation and subsequent proteasomal degradation of cyclin-dependent kinase inhibitor 1B (p27^Kip1^), thus causing the promotion of the S-G_2_/M transition of cell cycle progression [27]. In osteosarcoma cells, CaMKIIα increases the phosphorylation of T-lymphoma and metastasis gene 1 (Tiam1) and its membrane localization, and then activates ras-related C3 botulinum toxin substrate 1(Rac1), inhibits the expression of p21^CIP/KIP^ and leads to a cell cycle progression [21]. In HeLa cells, CaMKII phosphorylates cell division cycle 25c (cdc25c) and enhances G_2_/M transition. Treatment of a synchronous population of HeLa cells with KN-93 or the microinjection of AC3-I (a specific peptide inhibitor of CaMKII) resulted in a cell cycle block in G_2_ phase [28]. In breast cancer cells, CaMKII triggers the export of breast cancer susceptibility gene 1 (BRCA1) from nucleus and decreases the BRCA1-dependent expression of p21^CIP/KIP^ [29]. Moreover, CaMKII also can stimulate Cyclin D1 expression via NF-κB in breast cancer cells, which results in promoting of G_1_/S transition [30]. In addition to the promotion of cell cycle progression, CaMKII also can block the cell cycle of different cancers. CaMKII may suppress cell cycle progression by stabilization of p53 via CaMKII-dependent phosphorylation of the RING-H2 type E3 ligase (Pirh2) in breast and lung cancer cell lines [31].

### Invasion and metastasis

More and more evidences support the critical role of CaMKII in cancer invasion and metastasis. Daft et al. [32] demonstrated that the upregulation of CaMKIIα was found in primary osteosarcoma tissues from patients and in aggressive osteosarcoma cell lines. The knockdown of CaMKIIα decreases motility and invasion, whereas CaMKIIα overexpression increases the tumorigenic properties of osteosarcoma cell lines in vitro. Wang et al. [33] found that the activation of CaMKII significantly increased cell motility and the capacity of wound healing in prostate cancer cell lines. The rate of wound closure was decreased by 80% after inhibition of CaMKII. Bourguignon et al. [34] showed that CaMKII involved in hyaluronan (HA)-CD44-mediated signaling and modulated the adhesion and migration of head and neck squamous cell carcinoma (HNSCC) cells. HA-CD44 binding induces the leukemia-associated Rho guanine nucleotide exchange factor (LARG)-specific RhoA signaling and phospholipase Cε (PLCε) activity. The activation of RhoA- PLCε stimulates inositol 1,4,5-triphosphate production, intracellular Ca2+ mobilization, and subsequently activates CaMKII, leading to phosphorylation of the cytoskeletal protein, filamin. The phosphorylation of filamin reduces its interaction with filamentous actin, promoting HNSCC cell migration. Li et al. [35] reported that apoptosis regulatory protein Siva1 inhibited stathmin's activity through CaMKII dependent phosphorylation of stathmin at Ser16 in breast cancer cells. The formation of microtubules and impedes focal adhesion assembly, cell migration, and epithelial–mesenchymal transition (EMT) were enhanced by Siva1 via the inhibition of stathmin. Cuddapah et al. [36] indicated that the interaction of ClC-3 and CaMKII played critical role in the invasion and metastasis properties of glioma cells. CaMKII phosphorylated ClC-3 at Ser109 and led to an increase in ClC-3 current. ClC-3 enhances migration of glioma cells, and pharmacological inhibition with NPPB2 demonstrates a requirement for chloride channels to support the glioma invasion. In addition, recent evidence elucidates that bradykinin increases human glioma cell motility. Bradykinin activates the bradykinin receptor B2 (B2R), leading to increases in Ca2+ and enhanced the migration of glioma cells. The action of bradykinin in glioma is related to Ca2+-activated Cl currents, which is modulated by CaMKII [37]. Umemura et al.[38] found another mechanism of CaMKII involving cancer cell invasion. They showed that store-operated Ca2+ entry (SOCE) promoted melanoma progression by enhancing cell invasion and metastasis through activation of Erk signaling via the CaMKII/Raf-1/Erk pathway, irrespective of the status of Braf. Najdi et al. [39] proposed that CaMKII involved in the auto-activating loop of Wnt and CaMKII, and skew the balance of T-cell factors (TCFs). Inappropriate activation of this signal pathway would contribute to the cause and aggressive behavior of colon cancer cells.

### Apoptosis

The anti-apoptotic activity of CaMKII was found in recent studies. One anti-apoptotic pathway utilized by CaMKII is inhibition of caspase expression at the procaspase level and inhibition of caspase activation. Overexpression of CaMKII resulted in inhibition of procaspase-7 and procaspase-8 expression. And the activation of caspase-2, -7, and -8 could be prevented or diminished by overexpression of CaMKII [40]. Cohen et al.[41] reported that downregulation of ErbB could suppress the CaMKII signaling, which is coincident with the induction of apoptosis in breast and prostate cancer cells.

## THE ROLE OF CaMKII AS A BIOMARKER IN CANCER DIAGNOSIS

The role of CaMKII as a biomarker in cancer diagnosis was evaluated in some studies. Epigenetic regulation of gene expression through changes in CpG methylation in the promoter plays pivotal roles during cancioigenesis. Kim et al. [42] found that normal breast cells and breast cancer cells had different CaMKIIβ promoter methylation status. Based on this result, they suggested the promoter methylations of CaMKIIβ can be used as a biomarker for the diagnosis of breast cancer. Mamaeva et al. [43] showed that the expression profile of CaMKII isoforms was tissue-specific and could be used as a biomarker to distinguish the origination of cancer cells. They examined the gene expression of the four isoforms of CaMKII: α, β, γ, and δ in prostate cancer cell line C4-2B, PC3, LNCaP, and DU145. Only C4-2B and PC-3 cells, which are both derived from bone metastases, express all four isoforms of CaMKII. In another study, expression profiling of primary tumor tissues from 12 colon and 12 rectal cancers was performed using oligonucleotide microarray analysis. Of the genes differentially expressed between colon and rectal cancer, CaMKIIγ was one of the most significantly altered. CaMKIIγ provides potential candidate for distinguishing between colon and rectal cancer [44]. In a recent study, Chai et al. [5] demonstrated that CaMKIIγ was aberrantly expressed in human NSCLC tissues and correlated well with the degree of malignancy, and CaMKIIγ could be used as a potential biomarker of malignancy for NSCLC. Cancer cachexia is a syndrome associated with malignant tumor disease defined by weight loss, asthenia and anorexia. Stephens et al. [45] indicated that CaMKIIβ directly involved in human cancer cachexia. The activation of CaMKIIβ appears to be a general marker of muscle wasting in human cancer cachexia.

## THE ROLE OF CaMKII IN CANCER THERAPY

### Therapy resistance

Cellular Fas-associating protein with a novel death domain (FADD)-like interleukin-1β-converting enzyme inhibitory protein (c-FLIP), contributes to cancer therapy resistance has been demonstrated in some studies [46-48]. Yang et al. reported that the expression and phosphorylation of c-FLIP_L_ proteins was regulated by CaMKII [49]. When CaMKII activity was inhibited, c-FLIP expression reduced and the resistance glioma cells became sensitive to Fas-mediated apoptosis [50]. The similar results was also found in melanoma cells. KN-93 could sensitize resistant melanoma cells to TNF-related apoptosis-inducing ligand (TRAIL)-induced apoptosis via downregulated of c-FLIP proteins [51]. In addition, Rodriguez-Mora et al. [52] indicated that CaMK inhibitor could increase the treatment efficacy of doxorubicin, ionizing radiation, or photodynamic therapy in breast cancer cells. CaMKII participated in hydrogen peroxide-induced Erk phosphorylation was considered to be the underlying mechanism. Riganti et al. [53] showed that the transient increase of Ca2+ may activate CaMKII, which in turn phosphorylated and activated the transcription factor hypoxia-inducible factors 1α (HIF-1α) in colon cancer cells. As a consequence of HIF-1α nuclear translocation, P-glycoprotein 1 is overexpressed, intracellular accumulation of doxorubicin is reduced and cytotoxic effects of doxorubicin are prevented. These studies propose the critical role of CaMKII in cancer therapy resistance and targeting these pathways may provide novel therapeutic strategies in treatment of cancer.

### Modulation of therapy efficiency

For patients with inoperable or disseminated neuroblastoma, one of the most actively employed treatment approaches is targeted radiotherapy using ^131^I-labeled metaiodobenzylguanidine (MIBG) [54]. The efficacy of ^131^I-MIBG therapy is related to norepinephrine transporter (NET), which activity is largely modulated through specific intracellular signaling cascades, and candidate pathways for its regulation including protein kinase C (PKC), mitogen activated protein kinase (MAPK), phosphatidyl inositol-3 kinase (PI3K), and CaMKII [55]. Melittin, a water-soluble 26-amino acid peptide derived from bee venom of Apis mellifera, can exert toxic or inhibitory effects on many types of cancer cells [56-58]. Wang et al. [59] demonstrated that melittin potentiated the TRAIL induced apoptotic effects in human hepatocellular carcinoma cells by activating the CaMKII- transforming growth factor-β-activated kinase 1 (TAK1)- c-Jun N-terminal kinase (JNK)/p38 pathway but inhibiting the IKK-NFκB pathway. Fucoidan, a fucose-rich polysaccharide, is isolated from brown seaweed such as Cladosiphon okamuranus and Fucus evanescens. Recent studies have reported its various biological activities including anti-inflammatory, anti-coagulant, and anti-cancer [60, 61]. Chen et al. [62] indicated that fucoidan treatment inhibited cell growth and induced apoptosis in breast and colon cancer though the promotion of endoplasmic reticulum (ER) Ca2+-dependent CaMKII phosphorylation. Additionally, fucoidan has been linked to the increase of ER-stress and release of Ca2+ intracellularly. Cytosolic Ca2+ binds to calmodulin to activate CaMKII signalling, leading to ER stress-induced cell apoptosis through activating the mitochondrial apoptosis pathway[63].

## CAMKII INHIBITORS

During last two decades, a number of CaMKII inhibitors have been synthesized or found [14, 64]. Current knowledge about CaMKII control on physiological or pathological functions is largely based on the researches on these inhibitors. The most widely used inhibitor for study of cellular and in vivo functions of CaMKII has been KN-93 and KN-62. KN-93 and KN-62 share the same structural elements and mechanism of action. Inhibition by both is competitive with Ca2+/CaM and not competitive with ATP [14]. KN-93 and KN-62 have been found to induce cell cycle arrest in a variety of cancer cells. The G_1_ and/or S phase arrest effect of KN-93 and KN-62 on K562 and HeLa cells has been reported [65, 66]. KN-93 decreased cyclin-dependent kinase (cdk) 4 activity by reducing Cyclin D1 levels and cdk2 activity by enhancing p27^Kip1^ expression, causing cell cycle blocked at the G_1_ phase [67]. However, one of the limitations of KN-93 and KN-62 is low potency and absence of highly specific inhibition. KN-93 and KN-62 cannot discriminate between CaMKII and CaMKIV and also inhibit voltage-gated K+ and Ca2+ channels [68, 69]. Another limitation of KN-93 and KN-62 is that these compounds inhibit CaMKII activity by interfering with their binding to the Ca2+/CaM complex. However, once CaMKII has become activated (autophosphorylated), its dependence on further Ca2+/CaM binding is markedly diminished [17]. Thus, these compounds exert considerably less inhibition on CaMKII once it has become activated (autophosphorylated) [23].

CaMKII inhibitor 1 (CaMKIIN1), a peptide composed of 78-amino acids, functions as a potent and specific inhibitor of CaMKII was investigated recently. Wang et al. [70] found that CaMKIIN1 could inhibit prostate cancer growth in vivo. The inhibition effect was attributed to the down-regulation of insulin-like growth factor 1 (IGF-1), ErbB2, and vascular endothelial growth factor (VEGF) downstream kinases PI3K/Akt, as well as the MEK/Erk-mediated signaling pathways. Cao et al. [8, 71] identified human CaMKII inhibitory proteins, hCaMKIINα and hCaMKIINβ, which directly interacted with activated CaMKII and effectively inhibits CaMKII activity. hCaMKIINα could inhibit human colon adenocarcinoma cell growth both in vitro and in vivo by arresting cell cycle at the S phase. The effect of hCaMKIINα on cell cycle is correlated with up-regulation of p27^Kip1^ expression, which may be due to the inhibition of proteasome degradation of p27^Kip1^. Moreover, hCaMKIINα may suppress the activity of MEK/Erk, which is prerequisite to the inhibition of Thr187 phosphorylation and subsequent proteasomal degradation of p27^Kip1^, causing the inhibition of S phase progression of cell cycle. For hCaMKIINβ, the cell cycle arrest effect was depended on the upregulation of p21^CIP/KIP^, p53, and B-cell lymphoma 2 (Bcl-2)-associated X protein (Bax) and downregulation of Cyclin A, Cyclin D1, Cyclin E, Cyclin-dependent kinase 2 (CDK2), phosphorylated Rb, and Bcl-2 [72].

Other CaMKII inhibitors include some small molecules such as bosutinib, berberine, and berbamine. In contrast to other CaMKII inhibitors, which interfere with Ca2+/CaM complex formation, bosutinib directly targets the adenosine triphosphate binding site of CaMKIIγ and exerts the CaMKII inhibition effect [73]. Berberine, which is an isoquinoline alkaloid, decreases phosphorylation of CaMKII and blocks subsequent MEK1 activation as well as p27^Kip1^ protein degradation in human hepatoma cells [74]. Berbamine is a structurally unique bisbenzylisoquinoline isolated from Berberis amurensis. It can specifically bind to the ATP binding pocket of CaMKII, inhibits its phosphorylation and triggers apoptosis of leukemia cells [75]. In addition, Berbamine could suppress the growth of liver cancer cells in vitro and in vivo through inhibited CAMKII phosphorylation and directly down-regulated CAMKII [76].

## CONCLUSIONS

Strong evidences now show that CaMKII has emerged as a key nodal protein in the modulation of cell proliferation, cell cycle, invasion and metastasis, and therapy efficacy in variety of malignant diseases. These data establish CaMKII as a novel therapeutic target whose modulation presents new opportunities for cancer treatment. In this review, we also describe the role of CaMKII in the diagnosis of different kinds of cancer and recent progress in the development of CaMKII inhibitors. Although our knowledge about CaMKII is advancing, major questions still remain. Due to the particularly elegant relationship between the structure and function of the kinase, CaMKII is able to translate a diverse set of signaling events into downstream physiological effects. If we hope to use this kinase deeply in cancer diagnosis and therapy, we also need improve the understanding of CaMKII activation mechanisms. Additionally, the development of clinically applicable CaMKII inhibitory drugs to test the clinical benefit of CaMKII inhibition is also very important in the future researches.
